# Correlates of condom use among female sex workers in The Gambia: results of a cross-sectional survey

**DOI:** 10.7717/peerj.1076

**Published:** 2015-08-06

**Authors:** Ashley L. Grosso, Esther L. Lei, Sosthenes C. Ketende, Sarah Peitzmeier, Krystal Mason, Nuha Ceesay, Daouda Diouf, Fatou Maria Drame, Jaegan Loum, Erin Papworth, Stefan Baral

**Affiliations:** 1 Department of Epidemiology, Center for Public Health and Human Rights, Johns Hopkins Bloomberg School of Public Health, Baltimore, MD, USA; 2 UNAIDS-The Gambia, Banjul, The Gambia; 3 Enda Sante, Dakar, Senegal; 4 Department of Geography, School of Social Sciences, Gaston Berger University, Saint-Louis, Senegal; 5 Network of AIDS Service Organizations, Banjul, The Gambia; 6 Department of Epidemiology, Center for Public Health and Human Rights, Johns Hopkins Bloomberg School of Public Health, Dakar, Senegal

**Keywords:** Condoms, Female sex workers, The Gambia

## Abstract

**Objectives.** This study examined correlates of condom use among 248 female sex workers (FSW) in The Gambia.

**Methods.** Between July and August 2011, FSW in The Gambia who were older than 16 years of age, the age of consent in The Gambia, were recruited for the study using venue-based sampling and snowball sampling, beginning with seeds who were established clients with the Network of AIDS Services Organizations. To be eligible, FSW must have reported selling sex for money, favors, or goods in the past 12 months. Bivariate and multivariate logistic regressions were used to determine associations and the relative odds of the independent variables with condom use. Four different condom use dependent variables were used: consistent condom use in the past six months during vaginal or anal sex with all clients and partners; consistent condom use in the past month during vaginal sex with new clients; consistent condom use in the past month during vaginal sex with nonpaying partners (including boyfriends, husbands, or casual sexual partners); and condom use at last vaginal or anal sex with a nonpaying partner.

**Results.** Many FSW (67.34%, *n* = 167) reported it was not at all difficult to negotiate condom use with clients in all applicable situations, and these FSW were more likely to report consistent condom use with all clients and partners in the past 6 months (aOR 3.47, 95% CI [1.70–7.07]) compared to those perceiving any difficulty in condom negotiation. In addition, FSW were more likely to report using condoms in the past month with new clients (aOR 8.04, 95% CI [2.11–30.65]) and in the past month with nonpaying partners (aOR 2.93, 95% CI [1.09–7.89]) if they had been tested for HIV in the past year. Women who bought all their condoms were less likely than those who received all of their condoms for free (aOR 0.38, 95% CI [0.15–0.97]) to have used a condom at last vaginal or anal sex with a nonpaying partner.

**Conclusions.** HIV and sexually transmitted infection (STI) prevention interventions for FSW should aim to improve condom negotiation self-efficacy since women who report less difficulty negotiating condom use are more likely to use condoms with clients. Interventions should also be aimed at structural issues such as increasing access to free condoms and HIV testing since these were positively associated with condom use among FSW.

## Introduction

In the small Western African country of The Gambia, approximately 14,000 people are living with HIV, including 7,700 women aged 15 and older. This results in an HIV prevalence of approximately 1.5% in the adult population (UNAIDS, 2011). However, data suggest the HIV epidemic in Western Africa, including The Gambia, is concentrated among key populations including female sex workers (FSW) (Lowndes et al., 2008; Papworth et al., 2013). HIV prevalence among FSW in The Gambia is estimated to be 15.9% (Peitzmeier et al., 2013). In addition, one study found that approximately 25% of FSW in The Gambia reported ulcers, sores, or genital discharge characteristic of a sexually transmitted infection (STI) in the past year (Jallow, 2011).

Consistent condom use has been shown to reduce the spread of HIV and other STIs (Alfonsi & Shlay, 2005; Varghese et al., 2002). A behavioral surveillance survey among FSW in The Gambia in 2010 found while most (96.7%) reported using a condom at last sex with clients, only a minority of FSW (15.6%) reported using a condom at last sex with nonpaying partners (Jallow, 2011). One study conducted in The Gambia from 1989–1990 also found that there was no significant association between condom use and age, education level, marital status, country of origin, number of children, or whether or not the FSW had a regular partner (Pickering et al., 1993).

Research throughout sub-Saharan Africa has found demographic variables (Kayembe et al., 2008), client-related factors (Oladosu & Ladipo, 2001), health system issues, violence (Stadler & Delany, 2006), and social capital are related to condom use among FSW with their clients and nonpaying partners (Fonner et al., 2014). Studies have found demographic variables such as age (Kayembe et al., 2008), education (Adu-Oppong et al., 2007), rural or urban location (Voeten et al., 2007), country of origin (Alary et al., 2002), and having children (Papworth et al., 2015) to be related to condom use among FSW. For example, age was found to be associated with consistent condom use in the past 30 days with all clients and partners among FSW in the Democratic Republic of the Congo, with those aged 20 to 44 years old more likely to report consistent condom use than those under age 20 (Kayembe et al., 2008). FSW between the ages of 40 and 50 in Senegal were more likely to use condoms consistently with their regular nonpaying partners than FSW aged 20–30 (Wang et al., 2007). In contrast, in Ghana younger age (13 to 24 years, compared to older than 24 years) was associated with consistent condom use among FSW (though the type of client/partner and time period were not specified) (Adu-Oppong et al., 2007). In the same study in Ghana, higher levels of education were positively associated with consistent condom use. In Kenya, FSW in rural areas reported a higher proportion of sex acts with all partners without a condom in the past two weeks than FSW in urban areas (Voeten et al., 2007). Country of origin was found to be associated with condom use among FSW in Benin, where FSW from Nigeria were more likely to report condom use with clients in the past week than FSW from Togo, Ghana, and Benin (Alary et al., 2002). Having one or more children was positively associated with consistent condom use with new clients in the past 30 days but negatively associated with consistent condom use with nonpaying partners in the past 30 days among FSW in Burkina Faso.

Client-related factors including suggesting the use of condoms to clients (Oladosu & Ladipo, 2001) and clients paying more not to use condoms (Adu-Oppong et al., 2007) have also been associated with condom use among FSW. Asking clients to use condoms was found to be positively associated with consistent condom use in the last five sex acts among FSW in Nigeria (Oladosu & Ladipo, 2001). In several settings including Zambia, the Democratic Republic of the Congo, Ghana, and Nigeria, FSW have reported having sex with clients without condoms for more money than sex with condoms (Adu-Oppong et al., 2007; Agha & Chulu Nchima, 2004; Ntumbanzondo et al., 2006; Umar, Adekunle & Bakare, 2001).

Access to health services is also related to condom use among FSW (Wang et al., 2007). In Senegal, prior HIV testing among FSW was negatively associated with consistent condom use with regular nonpaying partners (Wang et al., 2007), while in the Democratic Republic of the Congo prior HIV testing was positively associated with consistent condom use in the past 30 days with all clients and partners (Kayembe et al., 2008). Disclosure of sex work to a health worker may also affect condom access and use. In a qualitative study in Uganda, FSW cited criminalization of sex work as a barrier for the healthcare system to provide condoms to FSW (Scorgie et al., 2013). Receiving HIV prevention information may also be related to condom use. In Ghana, FSW who were more knowledgeable about HIV were more likely to use condoms consistently (Adu-Oppong et al., 2007). Additionally, lack of availability or access to free condoms has been cited as a reason for inconsistent condom use among FSW in Ghana (Adu-Oppong et al., 2007).

Violence toward FSW from clients, nonpaying partners, police, or others may be negatively related to condom use (Shannon & Csete, 2010). In South Africa, threats of violence from clients and violence from nonpaying partners have been cited by FSW as barriers to condom use (Stadler & Delany, 2006; Varga, 1997).

Social cohesion among FSW in Swaziland, including being able to count on FSW colleagues to support the use of condoms, was found to be positively related to consistent condom use with all clients and partners in the past week (Fonner et al., 2014).

There is a need to better characterize prevention strategies among FSW to inform increasing investments in targeted HIV and STI prevention programs for the FSW population globally (Kerrigan et al., 2013). Barrier protection strategies including condom use among FSW are core components of HIV and STI prevention intervention strategies. Thus this study aims to understand correlates of condom use among FSW in The Gambia. Prior literature on condom use among FSW informed the models included in this study.

## Methods

### Study design, population, and recruitment

The study was conducted in The Gambia at a private voluntary counseling and testing (VCT) site managed by the Network of AIDS Services Organizations (NASO). Enda-Santé, a nongovernmental organization with experience in HIV prevention service delivery for FSW populations, provided study staff with sensitivity training on research ethics for working with FSW. The Johns Hopkins Bloomberg School of Public Health and UNAIDS-The Gambia Country Office provided technical support.

Between July and August 2011, 251 FSW aged 16 years and older (the age of consent in The Gambia) were recruited for the study using venue-based sampling and snowball sampling. This began with seeds who were established clients with NASO. To be eligible, FSW must have met the age inclusion criteria and reported selling sex for money, favors, or goods in the past 12 months.

### Procedure

The National Scientific and Ethics Committee in The Gambia and Johns Hopkins Bloomberg School of Public Health Institutional Review Board approved this study prior to recruitment in 2011 (IRB00003741 and for continued analysis IRB00005349). Participants were assigned a unique identifying code, taken into a private room with a trained staff member, assessed for eligibility, and then completed the oral voluntary consent process and a 45 minutes confidential interviewer-administered survey. The survey included modules characterizing multiple levels of HIV risk as explained in the Modified Social Ecological Model (MSEM), which characterizes the various levels of HIV risk among key populations. These levels include the individual, network, community, public policy, and prevalence or incidence of HIV in country (or epidemic stage) (Baral et al., 2013). The survey included questions on demographic information, exposure to human rights violations and discrimination, personal history, clients, mental health, knowledge and behaviors, difficulty suggesting condom use, social capital, and reproductive health.

Upon the completion of the survey, FSW received pre-test counseling for HIV and syphilis testing from a trained nurse or counselor. A nurse or on-site phlebotomist then administered the HIV rapid test. Participants were encouraged to wait 20 minutes for results and post-test counseling, but could leave earlier if desired. Participants testing positive for HIV or syphilis were referred to a nearby clinic for treatment. For indeterminate tests, the samples were sent to the Gambian National Public Health Laboratory, and participants could receive post-test counseling two weeks later using their unique identifier code. Participants received a travel reimbursement of 200 Dalasi (about $5) and were offered the opportunity to recruit other participants into the study. They were not remunerated for recruiting others or participating in the study.

### Measures

Four condom use outcome variables were used in the analysis for this paper. The first outcome, condom use in the past six months, is a composite variable created from two categorical survey questions on ever not using a condom in the past six months and frequency of condom use in the past six months. This composite variable was considered a more consistent measure, given possible social desirability bias and over-reporting of condom use. For the 14 participants whose answers were contradictory, the composite variable was coded 0 = did not always use condoms.

Condom use in the past month was also examined for information about more recent sexual practices. Since condom use varied by partner type (Peitzmeier et al., 2013), condom use in the past month with new clients served as the second condom use outcome and with nonpaying partners in the past month as the third outcome. Nonpaying partners were explained to participants as “partners who you have sex with, but are not your clients and therefore do not pay you for sex. This may include partners you live with, boyfriends or girlfriends (who do not pay you) and husbands. This would also include partners you had sex with for pleasure, without any sort of payment.” Due to the potential for recall bias, condom use at last vaginal or anal sex with a nonpaying partner was also assessed as the fourth and final outcome variable.

### Data analysis

Women who reported no new clients in the past 30 days were excluded from the analysis for outcome 2 (always using condoms with new clients in the past month).^1^1Fifty-three participants whose response to the question about the number of clients in the past 30 days was not recorded, no response, or don’t know answered the question about condom use with new clients in the past 30 days and were included in the analysis. For 4 participants who reported 1 or more new clients, no response was recorded for the question about condom use with new clients in the past 30 days, and they were excluded from the analysis. Women who reported no nonpaying partners in the past 30 days were excluded from the analysis for outcomes 3 and 4.^2^2Four participants whose response to the question about the number of nonpaying partners in the past 30 days was 0, not recorded, no response, or don’t know answered the question about condom use with nonpaying partners in the past 30 days and were included in the analysis. Two participants who reported 1 or more nonpaying partners responded “don’t know” to the question about condom use with nonpaying partners in the past 30 days and were excluded from the analysis. Three participants whose response to the question about the number of nonpaying partners in the past 30 days was 0, not recorded, or don’t know answered the question about condom use with nonpaying partners at last sex and were included in the analysis. Bivariate logistic regressions were used to determine estimates of the associations between the covariates and the outcome of interest, while multivariate logistic regressions were used to determine adjusted associations of covariates with the outcomes. The choice of covariates was informed by previous literature (as described in the introduction), statistical significance in bivariate logistic regressions at *p* < 0.05, amount of missing data for each variable, and collinearity with other variables. Ultimately these covariates included FSW age, HIV status, education level, where the FSW grew up (rural Gambia, urban Gambia, or outside of Gambia), number of children, living arrangement, ever pregnant, difficulty suggesting condom use, condom acquisition, and other factors related to HIV/STI testing, violence, and stigma (See Table 2). For the independent variable on difficulty suggesting condom use with clients, a composite variable was created from eight questions on different scenarios listed in Table 1. Answers ranged from 0 = “very difficult” to 4 =“not at all difficult.” The composite variable was created by a score of 1 for each question to which a to which a participant responded “not at all difficult” and 0 otherwise, adding these scores, dividing by the number of questions that were applicable for that FSW, and multiplying by 100 to obtain a percentage. For instance, if participants answered seven out of eight questions “not at all difficult” and the remaining question “not applicable”, the variable was coded as 100.

**Table 1:** Characteristics of female sex workers in The Gambia.

Variable	Proportion (*n*)	Did not always use condoms past 6 months	Always used condoms past 6 months	Did not always use condoms in past month with new clients	Always used condoms in past month with new clients	Did not always use condoms in past month with nonpaying partners	Always used condoms in past month with nonpaying partners	Did not use a condom at last sex with a nonpaying partner	Used a condom at last sex with a nonpaying partner
**Age (years), range (mean)**	17–51 (31)	17–49 (31)	17–51 (31)	19–40 (29)	17–49 (31)	17–48 (30)	17–49 (31)	17–48 (30)	17–49 (31)
**Living with HIV**	15.9% (40/251)	17.6%	15.0%	31.3%	13.8%	19.8%	23.7%	20.9%	22.6%
**Education**									
None	30.0% (74/247)	33.0%	28.3%	25.0%	27.8%	32.1%	32.4%	32.8%	30.8%
Primary	30.4% (75/247)	25.0%	33.3%	37.5%	29.2%	30.9%	29.7%	29.9%	30.8%
Secondary +	39.6% (98/247)	42.1%	38.4%	37.5%	43.1%	37.0%	37.8%	37.3%	38.5%
**Grew up**									
Rural Gambia	16.3% (40/246)	23.4%	12.0%	50.0%	16.8%	17.5%	10.5%	60.6%	54.7%
Urban Gambia	47.6% (117/246)	46.6%	48.1%	18.8%	58.7%	57.5%	57.9%	16.7%	13.2%
Outside Gambia	36.2% (89/246)	29.6%	39.9%	31.3%	24.5%	25.0%	31.6%	22.7%	32.1%
**Marital status**									
Married	1.2% (3/248)	2.3%	0.6%	0.0%	1.4%	1.2%	2.6%	0.0%	3.8%
Cohabitating	0.8% (2/248)	2.3%	0.0%	6.3%	0.0%	0.0%	2.6%	1.5%	1.9%
Widowed/divorced/ separated	69.8% (173/248)	70.8%	69.2%	56.3%	73.8%	67.9%	60.5%	64.2%	66.0%
Single/never married	23.0% (57/248)	16.9%	26.4%	25.0%	20.0%	27.2%	29.0%	28.4%	24.5%
Other	5.2% (13/248)	7.9%	3.8%	12.5%	4.8%	3.7%	5.3%	6.0%	3.8%
**Number of children**									
Childless	19.3% (46/238)	18.8%	19.6%	26.7%	17.0%	19.0%	27.8%	20.0%	25.5%
1–3	63.9% (152/238)	62.4%	64.7%	66.7%	63.8%	62.0%	55.6%	58.5%	60.8%
4 +	16.8% (40/238)	18.8%	15.7%	6.7%	19.2%	19.0%	16.7%	21.5%	13.7%
**Lives with**									
No one	33.1% (82/248)	25.9%	37.1%	6.3%	31.9%	30.0%	34.2%	31.8%	28.3%
Sexual partner/boyfriend	19.0% (47/248)	27.0%	14.5%	18.8%	19.4%	22.5%	18.4%	22.7%	20.8%
Other	46.1% (119/248)	47.2%	48.4%	75.0%	48.6%	47.5%	47.4%	45.5%	50.9%
**Non-paying partners in the past 30 days**									
Zero	48.9% (112/229)	35.7%	56.6%	33.3%	43.4%	1.3%	0.0%	1.5%	0.0%
One	47.2% (108/229)	60.7%	39.3%	46.7%	54.6%	93.8%	91.7%	92.5%	90.2%
Two	3.5% (8/229)	3.6%	3.5%	20.0%	1.4%	3.8%	8.3%	4.5%	9.8%
Four	0.4% (1/229)	0.0%	0.7%	0.0%	0.7%	1.3%	0.0%	1.5%	0.0%
**Reported having sold sex in the past 30 days**	85.3% (139/163)	96.7%	86.9%	100.0%	100.0%	98.8%	94.7%	98.5%	96.2%
**Number of regular clients reported in the past 30 days**									
Zero	25.8% (50/194)	15.3%	32.0%	25.0%	19.0%	8.1%	23.5%	9.6%	18.2%
One	10.8% (21/194)	11.1%	10.7%	0.0%	9.1%	12.9%	14.7%	11.5%	13.6%
Two	11.3% (22/194)	11.1%	11.5%	16.7%	8.3%	12.9%	17.7%	11.5%	20.5%
Three	10.3% (20/194)	6.9%	12.3%	8.3%	12.4%	11.3%	8.8%	13.5%	6.8%
≥ Four	41.8% (81/194)	55.6%	33.6%	50.0%	51.2%	54.8%	35.3%	53.9%	40.9%
**Number of new clients reported in the past 30 days**									
Zero	36.7% (65/177)	22.1%	45.9%	0.0%	0.0%	15.5%	35.3%	14.9%	31.8%
One	3.4% (6/177)	1.5%	4.6%	10.0%	5.1%	0.0%	11.8%	2.1%	4.6%
Two	6.8% (12/177)	5.9%	7.3%	20.0%	10.2%	6.9%	8.8%	6.4%	9.1%
Three	7.9% (14/177)	5.9%	9.2%	0.0%	14.3%	5.2%	17.7%	6.4%	13.6%
≥ Four	45.2% (80/177)	64.7%	33.0%	70.0%	70.4%	72.4%	26.5%	70.2%	40.9%
**Because of selling sex …**									
Afraid to seek healthcare	10.0% (25/249)	16.9%	6.3%	6.3%	11.7%	9.9%	2.6%	10.5%	3.8%
Tortured (by any perpetrator)	32.7% (81/248)	33.0%	32.5%	31.3%	34.0%	35.8%	40.5%	29.9%	46.2%
Police refused protection	24.9% (62/249)	23.6%	25.6%	12.5%	31.0%	33.3%	15.8%	29.9%	24.5%
Experienced verbal or physical harassment	38.6% (96/249)	38.2%	38.8%	31.3%	51.0%	54.3%	23.7%	56.7%	28.3%
Blackmailed (by any perpetrator)	24.5% (61/249)	22.5%	25.6%	12.5%	20.0%	18.5%	34.2%	13.4%	37.7%
Beaten up (by any perpetrator)	20.9% (50/239)	26.7%	17.7%	13.3%	20.7%	28.4%	29.7%	22.4%	36.5%
**Safe places to socialize with sex workers**	79.8% (198/248)	76.4%	81.8%	56.3%	81.3%	84.0%	86.8%	83.6%	84.9%
**A family member knows she sells sex**	13.3% (33/249)	27.0%	21.9%	31.3%	29.7%	30.9%	31.6%	32.8%	30.2%
**Disclosed sex work to healthcare worker**	33.5% (83/249)	37.1%	31.5%	12.5%	45.1%	53.1%	21.1%	56.7%	26.4%
**Ever offered more money for unprotected sex**	42.2% (102/242)	43.0%	41.7%	31.3%	51.4%	60.5%	37.8%	58.2%	45.3%
**Tested for STIs in past year**	45.3% (112/247)	37.5%	49.7%	31.3%	53.9%	43.2%	47.2%	47.8%	41.2%
**Tested for HIV in past year**	75.6% (183/242)	67.8%	80.0%	33.3%	71.6%	57.7%	79.0%	57.8%	73.6%
**Received HIV prevention information in past year**	65.8% (156/237)	51.2%	74.2%	25.0%	62.9%	61.3%	76.3%	60.6%	71.7%
**Ever pregnant**	78.9% (194/246)	71.6%	82.9%	62.5%	77.1%	86.4%	68.4%	85.1%	73.6%
**Can count on sex worker colleagues to support condom use**									
Strongly disagree	11.7% (29/248)	19.1%	7.6%	18.8%	13.9%	13.6%	10.5%	13.4%	9.4%
Disagree	5.2% (12/248)	7.9%	3.8%	6.3%	5.6%	3.7%	7.9%	6.0%	5.7%
Agree	65.7% (163/248)	55.1%	71.7%	50.0%	64.6%	64.2%	65.8%	62.7%	67.9%
Strongly agree	17.3% (43/248)	18.0%	17.0%	25.0%	16.0%	18.5%	15.8%	17.9%	17.0%
**Condom acquisition**									
Get all for free	19.5% (46/236)	19.8%	19.3%	18.8%	16.0%	8.8%	27.0%	9.1%	23.1%
Buy all	69.5% (164/236)	64.0%	72.7%	75.0%	68.1%	75.0%	64.9%	74.2%	67.3%
Buy and get for free	11.0% (26/236)	16.3%	8.0%	6.3%	16.0%	16.3%	8.1%	16.7%	9.6%
**Difficulty suggesting condom use with clients**									
**Participants answering “not at all difficult” to survey questions used to create the “ease suggesting condom use with clients” composite variable**									
How difficult is it for you to suggest using condoms with a client, even if it might make him think that you have a sexually transmitted disease?	80.8% (198/245)	69.0%	87.3%	75.0%	81.1%	87.5%	86.8%	86.4%	86.8%
How difficult is it for you to insist on condom use if a client does not want to use one?	82.2% (203/247)	69.3%	89.3%	80.0%	82.1%	82.7%	86.5%	82.1%	84.6%
How difficult is it for you to continue to insist on condom use with a client even if he gets angry when you suggest it?	83.8% (207/247)	71.9%	90.5%	75.0%	84.8%	85.2%	89.5%	85.1%	86.8%
How difficult is it for you to insist on condom use with a client when he has been drinking or using drugs?	83.8% (207/247)	71.9%	90.5%	68.8%	87.6%	87.7%	84.2%	85.1%	88.7%
How difficult is it for you to insist on condom use with a client when you have been drinking or using drugs?	82.4% (201/244)	73.5%	87.3%	66.7%	84.7%	84.8%	89.5%	83.1%	88.7%
How difficult is it for you to insist on condom use with a client if you are sexually attracted to him?	79.8% (197/247)	67.4%	86.7%	62.5%	82.1%	84.0%	86.8%	82.1%	86.8%
How difficult is it for you to insist on condom use with a client if he offers you more money not to use a condom?	83.0% (205/247)	73.9%	88.1%	66.7%	85.5%	88.8%	84.2%	86.4%	86.8%
How difficult is it for you to insist on condom use with a client with whom you haven’t always used condoms in the past?	83.7% (205/245)	74.7%	88.6%	50.0%	86.0%	88.6%	84.2%	84.6%	88.7%
Answered “not at all difficult” to all of the above	67.3% (167/248)	49.4%	77.4%	37.5%	69.0%	69.1%	81.6%	65.7%	81.1%
Ease suggesting condom use with clients scale, range (mean)	0–100 (82.3)	0–100 (71.2)	0–100 (88.5)	0–100 (66.8)	0–100 (84.1)	0–100 (85.9)	0–100 (86.2)	0–100 (84.0)	0–100 (87.1)
**Condom use**									
Always used condoms in the past 6 months	63.8% (160/251)	–	–	56.3%	77.8%	40.7%	84.2%	38.8%	73.6%
Always used condoms in the past month with new clients	90.1% (145/161)	84.1%	94.6%	–	–	88.4%	88.0%	89.3%	86.8%
Always used condoms in the past month with nonpaying partners	31.9% (38/119)	11.1%	49.2%	27.3%	26.5%	–	–	1.5%	69.2%
Used condom at last vaginal or anal sex with nonpaying partner	44.2% (53/120)	25.5%	60.0%	45.5%	39.8%	19.8%	97.3%	–	–

**Table 2:** Bivariate associations of condom use among female sex workers in The Gambia.

Variable	Always used condoms in past 6 months (*n* = 251)		Always used condoms in past month with new clients (*n* = 161)		Always used condoms in past month with nonpaying partners (*n* = 119)		Used condom at last vaginal or anal sex with nonpaying partner (*n* = 120)	
	OR	(95% CI)	OR	(95 % CI)	OR	(95% CI)	OR	(95% CI)
**Age**	1.01	(0.98, 1.05)	1.06	(0.98, 1.15)	1.02	(0.97, 1.08)	1.01	(0.96, 1.07)
**Living with HIV**	0.83	(0.41, 1.65)	0.35	(0.11, 01.12)	1.26	(0.50, 3.18)	1.11	(0.46, 2.65)
**Education**								
None	1.00		1.00		1.00		1.00	
Primary	1.55	(0.79, 3.07)	0.70	(0.18, 2.67)	0.95	(0.36, 2.56)	1.10	(0.44, 2.76)
Secondary +	1.06	(0.57, 1.98)	1.03	(0.27, 3.89)	1.01	(0.40, 2.57)	1.10	(0.46, 2.63)
**Grew up**								
Rural Gambia	1.00		1.00		1.00		1.00	
Urban Gambia	2.05	(0.99, 4.24)	9.33^**^	(2.30, 37.94)	1.67	(0.49, 5.68)	1.14	(0.39, 3.29)
Outside Gambia	2.68^*^	(1.24, 5.79)	2.33	(0.68, 8.00)	2.10	(0.56, 7.87)	1.78	(0.55, 5.67)
**Number of children**	0.99	(0.85, 1.17)	1.60^*^	(1.01, 2.53)	1.01	(0.80, 1.27)	0.95	(0.77, 1.19)
**Lives with**								
No one	1.00		1.00		1.00		1.00	
Sexual partner/boyfriend	0.37^*^	(0.18, 0.79)	0.20	(0.02, 2.05)	0.72	(0.24, 2.16)	1.03	(0.37, 2.85)
Other	0.71	(0.39, 1.32)	0.13	(0.02, 1.01)	0.87	(0.36, 2.10)	1.26	(0.54, 2.93)
**As a result of selling sex…**								
Afraid to seek healthcare	0.33^*^	(0.14, 0.77)	1.99	(0.25, 16.05)	0.25	(0.03, 2.05)	0.34	(0.07, 1.69)
Verbal or physical harassment (by any perpetrator)	1.02	(0.60, 1.74)	2.29	(0.76, 6.93)	0.26^*^	(0.11, 0.62)	0.30^**^	(0.14, 0.65)
Blackmailed (by any perpetrator)	1.19	(0.65, 2.19)	1.75	(0.38, 8.13)	2.29	(0.95, 5.48)	3.91^*^	(1.60, 9.56)
**Safe places to socialize with sex workers**	1.38	(0.73, 2.61)	3.37^*^	(1.15, 9.85)	1.26	(0.41, 3.84)	1.10	(0.41, 2.98)
**Can count on sex worker colleagues to support condom use**								
Strongly disagree	1.00		1.00		1.00		1.00	
Disagree	1.21	(0.33, 4.53)	1.20	(0.11, 13.32)	2.75	(0.38, 19.67)	1.35	(0.21, 8.62)
Agree	3.30^**^	(1.46, 7.42)	1.74	(0.42, 7.16)	1.32	(0.38, 4.57)	1.54	(0.47, 5.02)
Strongly agree	2.39	(0.91, 6.27)	0.86	(0.17, 4.33)	1.10	(0.25, 4.86)	1.35	(0.34, 5.44)
**Disclosed sex work to healthcare worker**	0.78	(0.45, 1.34)	5.76^*^	(1.26, 26.27)	0.24^*^	(0.10, 0.58)	0.27^**^	(0.13, 0.60)
**Tested for STIs past year**	1.65	(0.97, 2.80)	2.57	(0.85, 7.77)	1.18	(0.53, 2.59)	0.77	(0.37, 1.60)
**Tested for HIV in past year**	1.90^*^	(1.04, 3.45)	5.05^**^	(1.62, 15.70)	2.75^*^	(1.12, 6.76)	2.03	(0.93, 4.46)
**Received HIV prevention information in past year**	2.74^***^	(1.57, 4.79)	5.09 ^**^	(1.56, 16.60)	2.04	(0.85, 4.88)	1.65	(0.76, 3.58)
**Ever pregnant**	1.93^*^	(1.03, 3.58)	2.02	(0.68, 5.97)	0.34^*^	(0.13, 0.87)	0.49	(0.20, 1.21)
**Condom acquisition**								
Get all for free	1.00		1.00		1.00		1.00	
Buy all	1.16	(0.59, 2.30)	1.07	(0.28, 4.09)	0.28^*^	(0.10, 0.82)	0.36	(0.12, 1.04)
Buy and get for free	0.50	(0.19, 1.33)	3.00	(0.29, 31.01)	0.16^*^	(0.03, 0.79)	0.23^*^	(0.54, 0.96)
**Ever offered more money for non-condom-protected sex**	0.95	(0.56, 1.61)	2.33	(0.77, 7.04)	0.40^*^	(0.18, 0.88)	0.59	(0.29, 1.23)
**Ease suggesting condom use with clients**	1.02^***^	(1.01, 1.03)	1.02^*^	(1.00, 1.03)	–	–	–	–

**Notes.**

* *p* < 0.05.

** *p* < 0.01.

*** *p* < 0.001.

In order to compare between factors associated with condom use during different time periods and with different partners, variables that were significantly related to any of the four outcome variables in the bivariate analysis were included in the multivariate models. The only exception was the variable measuring difficulty suggesting condom use with clients, which was excluded from the multivariate models examining correlates of condom use with nonpaying partners. Data were analyzed using Stata version 13.1 (College Station, Texas).

## Results

Descriptive statistics of demographic characteristics and survey responses of FSW who participated are reported elsewhere (Peitzmeier et al., 2013) and summarized in Table 1. The mean age of participants was 31 years old, more than 60% had primary school education or less, and just under 40% were born in The Gambia (Peitzmeier et al., 2013). Out of 163 women responding, 14.7% (*n* = 24) reported not having sold sex at all in the past 30 days. In addition, out of 177 women responding, 36.7% (*n* = 65) reported not having sold sex to a new client in the past 30 days. Results of the bivariate analyses are reported in Table 2.

### Bivariate and multivariate analysis results

#### Consistent condom use in the past six months with all partners

As shown in Table 2, in the bivariate model those who said it was not at all difficult to suggest condom use with clients in more situations that applied to them were more likely to report consistent condom use in the past 6 months with all partners compared to those who found it not at all difficult in fewer situations (OR:1.02, 95% CI[1.01–1.03]). FSW who lived with a sexual partner or boyfriend (OR:0.37, 95% CI [0.18–0.79]) and were afraid to seek healthcare (OR:0.33, 95% CI [0.14–0.77]) were less likely to consistently use condoms in the past six months with all clients and partners. FSW who had tested for HIV in the past 12 months (OR:1.90, 95% CI[1.04–3.45]), had received HIV prevention information in the past 12 months (OR:2.74, 95% CI [1.57–4.79]), or had ever been pregnant (OR:1.93, 95% CI [1.03–3.58]) were more likely to consistently use condoms with all clients and partners in the past six months. In addition, FSW who felt that they could depend on other sex workers to support their use of condoms were more likely to use condoms with all clients and partners in the past six months (OR:3.30, 95% CI [1.46–7.42]). In the multivariate model, shown in Table 3, those who reported it was not at all difficult to suggest condom use in more situations had higher odds of consistent condom use in the past six months with all clients and partners (aOR:1.03, 95% CI [1.01–1.04]). Fear of seeking health services was negatively and independently associated with consistent condom use with all clients and partners in the past six months (aOR:0.26, 95% CI [0.07–0.88]).

**Table 3:** Multivariate models of factors associated with condom use among female sex workers in The Gambia.

	Always used condoms in past 6 months		Always used condoms in past month with new clients		Always used condoms in past month with nonpaying partners		Used condom at last vaginal or anal sex with nonpaying partner	
	aOR	95% CI	aOR	95% CI	aOR	95% CI	aOR	95% CI
**Grew up**								
Rural Gambia	REF	REF	REF	REF	REF	REF	REF	REF
Urban Gambia	0.40	0.14, 1.14	0.16	0.02, 1.28	0.08^*^	0.01, 0.64	0.30	0.06, 1.40
Outside Gambia	1.36	0.56, 3.29	0.52	0.06, 4.24	0.76	0.17, 3.39	1.85	0.49, 6.93
**Number of children**	0.94	0.75, 1.18	1.59	0.80, 3.18	1.02	0.71, 1.47	1.00	0.73, 1.37
**Lives with**								
No one	REF	REF	REF	REF	REF	REF	REF	REF
Sexual partner	0.69	0.24, 1.95	$\hat {}$	$\hat {}$	1.53	0.30, 7.67	1.35	0.30, 6.00
Other	0.94	0.43, 2.05	$\hat {}$	$\hat {}$	1.42	0.34, 5.95	1.95	0.60, 6.35
**Because of selling sex…**								
Afraid to seek healthcare	0.26^*^	0.07, 0.88	∼	∼	0.18	0.01, 4.25	0.36	0.05, 2.67
Experienced verbal or physical harassment	0.76	0.33, 2.09	1.57	0.19, 13.21	0.34	0.06, 1.92	0.31	0.74, 1.29
Experienced blackmail	0.73	0.33, 1.63	1.56	0.21, 11.90	2.04	0.55, 7.55	3.82^*^	1.20, 12.12
**Safe places to socialize with other sex workers**	0.77	0.30, 1.94	3.07	0.60, 15.61	0.51	0.08, 3.09	0.61	0.14, 2.66
**Can count on sex worker colleagues to support condom use**								
Strongly disagree	REF	REF	REF	REF	REF	REF	REF	REF
Disagree	1.19	0.21, 6.76	0.89	0.03, 23.19	8.28	0.47, 144.83	1.06	0.11, 10.37
Agree	1.95	0.59, 6.52	0.23	0.02, 3.50	1.08	0.73, 16.10	1.70	0.23, 11.74
Strongly agree	1.61	0.45, 5.78	0.33	0.02, 4.72	1.27	0.08, 19.51	1.52	0.21, 11.16
**Disclosed sex work to health worker**	1.15	0.53, 2.53	3.45	0.52, 22.90	0.47	0.11, 1.97	0.28^*^	0.09, 0.84
**Tested for HIV in past year**	1.64	0.76, 3.54	10.81^**^	1.90, 61.63	1.46	0.39, 5.46	1.14	0.40, 3.29
**Received HIV prevention information in past year**	1.76	0.78, 4.00	2.00	0.37, 10.75	7.11	0.97, 52.25	2.51	0.60, 10.44
**Ever pregnant**	0.60	0.19, 1.96	0.21	0.02, 2.75	0.09^*^	0.01, 0.79	0.57	0.13, 2.54
**Condom acquisition**								
Buy all	REF	REF	REF	REF	REF	REF	REF	REF
Get all for free	0.87	0.35, 2.18	2.80	0.28, 27.81	4.10	0.80, 21.00	3.75	0.89, 15.75
Buy and get for free	0.53	0.18, 1.56	1.63	0.14, 18.66	2.11	0.33, 13.42	2.69	0.59, 12.36
**Ever offered more money for non-condom-protected sex**	0.77	0.33, 1.79	0.64	0.10, 3.95	0.56	0.13, 2.33	1.93	0.56, 6.65
**Ease suggesting condom use with clients**	1.03^**^	1.01, 1.04	1.02	0.99, 1.06	–	–	–	–
Analysis sample (*n*)	207		142		106		108	

**Notes.**

95% confidence intervals in parentheses.

* *p* < 0.05.

** *p* < 0.01.

$\hat {}$ predicts success perfectly.

∼ omitted due to collinearity.

– omitted because questions did not include nonpaying partners.

#### Consistent condom use with new clients in the past month

FSW who grew up in an urban area in The Gambia were more likely to have consistently used condoms with new clients in the past month than those who grew up in a rural area in The Gambia (OR:9.33, 95% CI [2.30–37.94]). Number of children was positively associated with consistent condom use in the past month with new clients (OR:1.60, 95% CI [1.01–2.53]). Those who said suggesting condom use was not at all difficult in more situations were more likely to report consistent condom use in the past month with new clients (OR:1.02, 95% CI [1.00–1.03]). Condom use with new clients was also more likely among those who said there were safe places to socialize with other FSW (OR:3.37, 95% CI [1.15–9.85]), had disclosed involvement in sex work to a healthcare worker (OR:5.76, 95% CI [1.26–26.27]), or received HIV prevention information in the past year (OR:5.09, 95% CI [1.56–16.60]). HIV testing in the past 12 months was positively and significantly associated with consistent condom use with new clients in the past month in the bivariate (OR:5.05, 95% CI [1.62–15.70]) and multivariate (aOR:10.81, 95% CI [1.90–61.63]) models.

#### Consistent condom use with nonpaying partners in the past month

Verbal or physical harassment was negatively associated with consistent condom use in the past month with nonpaying partners (OR:0.26, 95% CI [0.11–0.62]). Those who disclosed involvement in sex work to a health worker were less likely to report consistent condom use with nonpaying partners in the past month (OR:0.24, 95% CI [0.10–0.58]), while those who tested for HIV in the past 12 months were more likely (OR:2.75, 95% CI [1.12–6.76]). Being offered more money for sex without a condom ever was negatively associated with consistent condom use with nonpaying partners in the past month (OR:0.40, 95% CI [0.18–0.88]). FSW who were ever pregnant had lower odds of consistent condom use in the past month with nonpaying partners (OR:0.34, 95% CI [0.13–0.87]). Women who bought all their condoms (OR:0.28, 95% CI [0.10–0.82]) or bought some and received some for free (OR:0.16, 95% CI [0.03–0.79]) were less likely to have consistently used condoms with nonpaying partners in the past month than those who received all their condoms for free. In the multivariate analysis, growing up in an urban area (aOR:0.08, 95% CI [0.01–0.64]) and ever being pregnant were negatively associated (aOR:0.09, 95% CI [0.01–0.79]) with consistent condom use in the past month with nonpaying partners.

#### Condom use at last vaginal or anal sex with a nonpaying partner

In the bivariate analysis, women were less likely to have used a condom at last sex with nonpaying partners if they had been verbally/physically harassed (OR:0.30, 95% CI [0.14–0.65]) but were more likely to have used condoms if they were blackmailed (OR:3.91, 95% CI [1.60–9.56]). Disclosing sex work to a health worker was negatively associated with condom use at last sex with a nonpaying partner (OR:0.27, 95% CI [0.13–0.60]). FSW who bought condoms and received them for free were less likely to have used a condom at last sex than those who received all their condoms for free (OR:0.23, 95% CI [0.54–0.96]). In the multivariate model, FSW who had been blackmailed had over three times higher odds of using a condom at last sex with a nonpaying partner (aOR:3.82, 95% CI [1.20–12.12]). Disclosing involvement in sex work to a health worker was negatively and independently associated with condom use at last sex with a nonpaying partner (aOR:0.28, 95% CI [0.09–0.84]) in the multivariate analysis.

## Discussion

This study adds to the literature on condom use among FSW by assessing correlates for condom use outcomes for three time points and differentiating between new clients and nonpaying partners. Health service factors emerged as correlates of condom use, and there were different factors associated with condom use by type of partner and time period.

Fear of seeking health services was negatively associated with consistent condom use in the past six months with all types of partners. FSW who do not use condoms with all partners may fear seeking health services because they may find out they have HIV or an STI or may fear stigma in the health setting. Another interpretation is that FSW who are afraid to seek health services may not receive counseling from health workers that would lead them to consistently use condoms. This explanation is supported by the finding that FSW who tested for HIV in the past year were more than ten times as likely to have consistently used condoms with new clients in the past month. This finding is similar to that of a study in the Democratic Republic of the Congo where prior HIV testing was positively associated with always using condoms in the past 30 days with all partners and clients (Kayembe et al., 2008). It is possible that pre- or posttest counseling led FSW to use condoms consistently with new clients, or learning their HIV status motivated them to protect themselves and their clients.

In contrast, disclosing involvement in sex work to a health worker was negatively associated with condom use at last sex with a nonpaying partner, and FSW who were ever pregnant were less likely to always use condoms in the past month with nonpaying partners. Taken together, these findings may reflect different reproductive goals with nonpaying partners than with clients (Papworth et al., 2015; Schwartz et al., 2015).

Because this study found several barriers to condom use by FSW, interventions should also be directed toward clients and nonpaying partners. Though there have been some studies of condom use by clients of FSW in other settings including the Dominican Republic (Barrington et al., 2009) and nonpaying partners of FSW in Benin (Lowndes et al., 2000), there have been relatively few interventions for clients and partners aimed at increasing condom use. A notable exception was an intervention for clients of FSW in Benin that included peer outreach, risk reduction counseling, distribution of condoms and information, and demonstrations of correct condom use (Lowndes et al., 2007). This type of intervention could also be considered for adaptation in The Gambia.

Due to limited timeline and budget, participants were recruited in urban areas using snowball sampling. The use of such methods can cause findings to have limited generalizability, although the relationships between variables in the sample still hold. In addition, responses were gauged using self-report questionnaires and inherently rely on the accuracy and completeness of participants’ responses. As such, social desirability and recall bias may be present. There were several differences in findings by time period and type of partner. Some of these differences may be due to recall bias. Condom use at last sex with a nonpaying partner may have been reported more accurately than consistent condom use in the past six months with all clients and partners. If FSW overestimated their condom use, the estimates may be biased away from the null. Because of this, the relationships between the independent variables and the outcomes should be interpreted more cautiously for the models examining condom use over longer time periods than for those examining condom use in more recent time periods. Bias may have also been introduced through limitations in data collection. For example, nonpaying partners were defined for participants as all partners who did not pay for sex. Separate data collection was not conducted on the varying partner types for nonpaying partners, such as steady/main partners, nonpaying clients, or one-time nonpaying partners.

Because of the structure of the questionnaire, two of the dependent variables referred to condom use during anal and vaginal sex, while other questions referred specifically to either vaginal, anal, or oral sex only. Due to missing data and a large number of “not applicable” responses, it was not possible to analyze the specific associations between difficulty suggesting condom use and reported actual condom use during anal and oral sex.

The questionnaire asked about regular (more than one-time) clients, but these questions were not applicable for about 87% of participants. Because these analyses are post-hoc, the sample size was not calculated to assess the relationships in this paper. Finally, because this study is cross-sectional, causality cannot be determined. More formative research is required to provide both causality and a qualitative context to the findings. Despite these limitations, this study has found evidence of the individual and social factors associated with condom use with new clients and non-paying partners among FSW in The Gambia.

## Conclusion

This study describes the current FSW situation in The Gambia related to condom use and lays the foundation for future programs and research. HIV and STI prevention, treatment and care service packages are needed for FSW. A menu-driven approach with tailored services is necessary due to diversity within this population (Kerrigan et al., 2013). Some FSW have difficulty or may not want to use condoms in some situations and with some types of partners. Because of this, additional prevention modes such as pre/post-exposure prophylaxis, and treatment for those living with HIV (FSW, their clients and partners) can provide protection against acquisition and transmission of HIV and/or STI to or from clients or nonpaying partners. Because there are relatively few studies of pre-exposure prophylaxis among FSW (Mutua et al., 2012; Singh & Mills, 2005; Vissers et al., 2008) more research is warranted. Decreasing fear of seeking health services, increasing provision and uptake of voluntary HIV counseling and testing, and empowering FSW to suggest condom use with clients may be strategies for increasing consistent condom use with clients. Health workers should be trained to build their clinical and cultural competence to provide services to FSW. This may reduce fear of seeking health services among FSW. Additionally, if FSW disclose their occupation to a health worker this may provide an opportunity for a discussion about safer sexual and conception practices with nonpaying partners.

## Supplemental Information

10.7717/peerj.1076/supp-1 Supplemental Information 1 Gambia FSW dataset

10.7717/peerj.1076/supp-2 Supplemental Information 2 Oral consent script
