# Supplementary material for: Correlates of condom use among female sex workers in The Gambia: results of a cross-sectional survey
Source: PeerJ. 2015 Aug 6;3:e1076. doi: 10.7717/peerj.1076 (PMC4540013; doi:10.7717/peerj.1076)
Supplement: Supplemental Information 2 [file peerj-03-1076-s002.doc]

#### **ORAL CONSENT SCRIPT**

**Consent Form for Interviews for Sex Workers**

**Study Title:** An Integrated Bio-Behavioral Survey of Most At Risk Populations (MARPS) including Female Sex Workers (FSW) and Men who have Sex with Men (MSM) in The Gambia

**Principal Investigator:** Stefan Baral

**IRB No.: 00003741**

**PI Version Date: Version 2, 7/19/11**

**Introduction**

This consent form explains the research study you are being asked to join. I will read and review this form with you now and give you an opportunity to ask any questions about the study before you agree to join the study. You may also ask questions at any time after you join the study.

**Purpose**

You are invited to take part in a research study. The purpose of this study is to learn about the experiences of sex workers, and improve services for sex workers. To join the study is voluntary. You may refuse to join, or you may withdraw your consent to be in the study, for any reason, without penalty.

**Why you are being asked to participate**

You are being invited to participate in this study because you reported exchanging sex for money in the last 12 months and you are 16 years of age or older.

**Procedures**

We are asking individuals who identify as sex workers to participate in a survey, HIV test and syphilis test. This includes participating in an HIV and Syphilis rapid test which includes pre- and optional post-test counseling. If found positive for HIV, we will provide counseling and referrals for treatment. If found positive for Syphilis, we will provide counseling and treatment for this infection. We will conduct one face-to-face survey with you. The survey would take place in a private room. We expect that the survey will take approximately 60 minutes of your time. Also, the rapid tests for HIV and Syphilis will take an estimated 20 additional minutes. We estimate that study participation will take approximately 90 minutes plus travel to the study site. If you choose to recruit others, we expect that it will take approximately 5 minutes to inform your contact about the study as well as the travel time needed to meet your colleague.

In the survey, we will ask you questions about your background, attitudes toward HIV, behaviors, and past experiences related to sex work and HIV. You do not have to answer any question that you feel uncomfortable with. We will also ask you to recruit additional individuals who identify as sex workers.

**Risks/Discomforts**

We do not think that being part of this study will create significant risks for you. In the survey I will be asking you questions about your day-to-day experience. Some of the topics may be emotional or difficult to discuss. You do not have to answer any questions that you would prefer not to answer. If you feel that you would like to speak to someone after the survey we can refer you to appropriate services.

There is a risk that if somebody finds out that you are participating in this study that they will discover that you are a sex worker. We will do everything we can to protect this information. Your name will not be collected at any point during this study.

Whether or not you decide to participate in the study will not affect your involvement in any other research study or any of the services you currently receive. You can stop the study at any time without penalty.

**Benefits**

There is no direct benefit to you from participating in this study. However, we hope the findings will help create HIV prevention, care and treatment programs that better meet the needs of sex workers. You may also benefit from having the opportunity to reflect on your experiences in a supportive and private environment.

**Reimbursement**

You will receive 200 Dalasis upon completion of the first interview as compensation for your time. We will also reimburse you for any travel expenses related to coming for the interview. We will also ask your help recruiting additional participants.

**Protecting data confidentiality**

No identifying information will be collected. Survey data will be identified only by a unique ID number.

**Voluntariness**

Your participation in this research project is completely voluntary. You have the right to withdraw from the research study at any time. You should ask the local study coordinator, Jaegan Loum at #9900254 if you have any questions about this research study. You may ask him questions in the future if you do not understand something that is being done.

**Who do I call if I have questions or problems?**

- Call the study coordinator, Jeagan Loum at 9900254 or principal investigator, Bai Cham 9902653 if you have any questions or concerns.

**Do you agree to take this one-time behavioral survey along with the HIV and syphilis rapid tests?**

**YES** _____ (interviewer signs bottom and continues to survey)

**NO**  _____ (interview stops)

**Interviewer Signature _____________________________ Date ___________**

**(If participant consents, interviewer will sign and date, if no consent, interview stops and thanks participant for their time)**
